# Comparative analysis of oral and intraperitoneal glucose tolerance tests in mice

**DOI:** 10.1016/j.molmet.2022.101440

**Published:** 2022-01-11

**Authors:** Lewin Small, Amy Ehrlich, Jo Iversen, Stephen P. Ashcroft, Kajetan Trošt, Thomas Moritz, Bolette Hartmann, Jens J. Holst, Jonas T. Treebak, Juleen R. Zierath, Romain Barrès

**Affiliations:** 1 Novo Nordisk Foundation Center for Basic Metabolic Research, Faculty of Health and Medical Sciences, University of Copenhagen, Denmark; 2 Swedish Metabolomics Centre, Department of Plant Physiology and Forest Genetics, Swedish University of Agricultural Sciences, Sweden; 3 Department of Biomedical Sciences, Faculty of Health and Medical Sciences, University of Copenhagen, Denmark; 4 Department of Physiology and Pharmacology and Section for Integrative Physiology, Department of Molecular Medicine and Surgery and Karolinska Institutet, Sweden; 5 Institut de Pharmacologie Moléculaire et Cellulaire, Université Côte d'Azur and CNRS, France

**Keywords:** I.P., Intraperitoneal, GTT, Glucose tolerance test, oGTT, Oral glucose tolerance test, ipGTT, Intraperitoneal glucose tolerance test, siGTT, Stable isotope-labelled glucose tolerance test, DIO, Diet-induced obesity, ANOVA, Analysis of variance, HFD, High-fat diet., Glucose tolerance test, Intraperitoneal, Oral, Mouse, Insulin, Incretin

## Abstract

**Objective:**

The glucose tolerance test (GTT) is widely used in preclinical research to investigate glucose metabolism, but there is no standardised way to administer glucose. The aim of this study was to directly compare the effect of the route of glucose administration on glucose and insulin kinetics during a GTT in mice.

**Methods:**

A GTT was performed in lean male and female mice and obese male mice and glucose was administered via the oral or intraperitoneal (I.P.) route. Samples were collected frequently during the GTT to provide a full time-course of the insulin and glucose excursions. In another cohort of lean male mice, plasma concentrations of insulin, c-peptide, and incretin hormones were measured at early time points after glucose administration. A stable-isotope labelled GTT (SiGTT) was then performed to delineate the contribution of exogenous and endogenous glucose to glycemia during the GTT, comparing both methods of glucose administration. Finally, we present a method to easily measure insulin from small volumes of blood during a GTT by directly assaying whole-blood insulin using ELISA and show a good concordance between whole-blood and plasma insulin measurements.

**Results:**

We report that I.P. glucose administration results in an elevated blood glucose excursion and a largely absent elevation in blood insulin and plasma incretin hormones when compared to oral administration. Utilising stable-isotope labelled glucose, we demonstrate that the difference in glucose excursion between the two routes of administration is mainly due to the lack of suppression of glucose production in I.P. injected mice. Additionally, rates of exogenous glucose appearance into circulation were different between lean and obese mice after I.P., but not after oral glucose administration.

**Conclusion:**

Reflecting on these data, we suggest that careful consideration be given to the route of glucose administration when planning a GTT procedure in mice and that in most circumstances the oral route of glucose administration should be preferred over the I.P. route to avoid possible artifacts originating from a non-physiological route.

## Highlights

• Intraperitoneal glucose administration does not promote insulin secretion.
• Exogenous glucose appearance is delayed in obese mice after intraperitoneal administration.
• Hepatic glucose production is suppressed after administering oral not intraperitoneal glucose.
• Measuring insulin from whole blood is comparable to that from plasma.

## 1 Introduction

The glucose tolerance test (GTT) is an important tool to diagnose individuals with impaired glucose metabolism, a key characteristic of diabetes. Due to its prolific clinical use in the diagnosis of diabetes, the GTT protocol in humans is relatively standardised. In the most common version, the oral glucose tolerance test (oGTT), an individual receives a glucose drink containing 75 g of glucose after an overnight fast, and blood glucose is measured in the fasting state and following glucose administration (usually 2 h after administration of the glucose drink). Because of its wide utility and ease of use, the GTT has been adapted for use in rodent models to study whole-body glucose metabolism *in vivo*. However, unlike in the human situation, the GTT protocol in mice is not standardised and can vary widely between laboratories, leading to different conclusions on the effect of similar interventions on glucose metabolism. A previous study investigated several variables in the mouse GTT protocol including time of fasting, glucose load, and the route of glucose administration; in it, the investigators surveyed mouse GTTs in the previous year and found that the majority (73 of 100 mice surveyed) utilised the intraperitoneal (I.P.) injection of glucose [1]. During the preparation of this manuscript, we performed a similar survey on the route of glucose administration for a GTT in published mouse studies in which a GTT was performed. In half of these studies (45/93) I.P. delivery was exclusively used with no justification as to why this route was preferred over the oral route. Additionally, The International Mouse Phenotyping Consortium exclusively uses the ipGTT rather than the oGTT as a measure of whole-body glucose tolerance in mice [2].

Although there has been some comparison between I.P. and oral delivery of glucose during a GTT, with mice receiving glucose orally demonstrating a lower glucose excursion and corresponding higher insulin secretion [1,3], no studies appear to have rigorously compared the effect of the route of glucose administration on glucose and insulin dynamics. As I.P. glucose administration is a non-physiological route of administration, partly bypassing the gastrointestinal (GI) tract, we hypothesised that there may be important differences in whole-body glucose handling when comparing between oral and I.P glucose administration. To examine this question, we performed GTTs in lean and diet-induced obese (DIO) male mice and lean female mice in which glucose was given by I.P. injection or by oral gavage. During the GTT, blood glucose and insulin were frequently measured following glucose administration to provide a complete glucose and insulin excursion. Further, in a separate cohort of mice, levels of incretin hormones and c-peptide were measured at early time points following glucose administration. We also performed a stable isotope-labelled GTT (siGTT) in lean male mice to determine differences in endogenous and exogenous glucose handling during the GTT comparing between routes of glucose administration. Our data revealed substantial differences in glucose and insulin kinetics during the GTT depending on the route of glucose administration and bring in to question the physiological relevance of I.P. glucose administration as a means of interrogating glucose metabolism in mice.

## 2 Methods

### 2.1 Animals

Experiments involving mice were designed according to the EU Directive 2010/63/EU and ARRIVE guidelines, approved by the Danish Animal Experiments Expectorate (license number 2019-15-0201-01663), and performed according to local ethics committee guidelines. Male and female C57BL/6NTac mice and male DIO C57BL/6NTac mice (fed 60% high-fat diet (HFD), Research Diets #D12492, from 6-weeks of age for a total of 14–16 weeks of HFD feeding) were purchased from Taconic. Mice had *ad libitum* access to water and either chow (lean mice, Altromin diet #1310) or a 60% HFD (DIO mice, Research Diets #D12492). Mice were maintained at 22 °C ± 1 °C on a 12:12 light–dark cycle and housed in groups of 5 mice. The mice were culled the week following the 2nd GTT at either the start of their active phase or after a 6-hour fast. They were injected with 100 mg/kg of pentobarbital and after deep anaesthesia (complete lack of pedal reflex) blood was taken by cardiac puncture and the mice were culled by cervical dislocation.

### 2.2 Glucose tolerance test

Glucose tolerance tests were performed in all mice between 20 and 26 weeks of age in the conscious state. On the day prior to the glucose tolerance test, an MRI was performed to determine body composition (Minispec LF90 MRI scanner, Bruker). Mice were fasted for 6 h (food removed at 8:00, 2 h after lights turn on and GTT started at 14:00) and 2.5 g of glucose/kg of lean mass was delivered either by I.P. injection or oral gavage of a 25% glucose solution dissolved in sterile saline. Dosage based on lean mass was performed due to the low rate of glucose disposal in adipose tissue, as previously discussed [3,4]. Blood glucose levels were monitored from the tail-tip using a hand-held glucometer (Contour XT, Bayer) in the basal state and 5, 10, 15, 20, 30, 45, 60, and 90 min following glucose administration. Whole blood insulin levels were determined in the basal state and 5, 10, 15, 20, 30, 45, and 60 min following glucose administration. Route of delivery was alternated between I.P. and oral delivery and each mouse received the other route of delivery for the following GTT so that each mouse had undergone both an I.P. and oral GTT. For the second GTT, 25 μCi/ml [U–^14^C] glucose (Perkin Elmer) was included in the glucose solution and 5 μl of blood was collected into tubes containing 50 μl of 0.3 M ZnSO_4_ in the basal state and 5, 10, 15, 20, and 30 min following glucose administration. Blood proteins were precipitated by the addition of 50 μl of 0.3 M Ba(OH)_2_, samples were spun at 10,000 *g* for 10 min before radioactivity of the supernatant was counted in a beta-counter after the addition of 3 ml of liquid scintillation cocktail (Ultima Gold, Perkin Elmer). For the measurement of plasma c-peptide, insulin, and incretin hormones (shown in Figure 2), forty 14-week-old male C57BL/6NTac mice were randomised into 5 groups (baseline, I.P. 5 min, I.P. 10 min, oral 5 min, oral 10 min), fasted for 6 h and then culled by cervical dislocation and their blood was immediately collected by cardiac puncture at baseline and 5 or 10 min following I.P. injection or oral gavage of 50 mg of glucose dissolved in 200 μl of sterile saline. Blood was collected into ethylenediaminetetraacetic acid (EDTA) tubes on ice, spun at 1,000 g at 4 °C for 10 min, and the obtained plasma was immediately frozen on dry ice.

**Figure 1:**
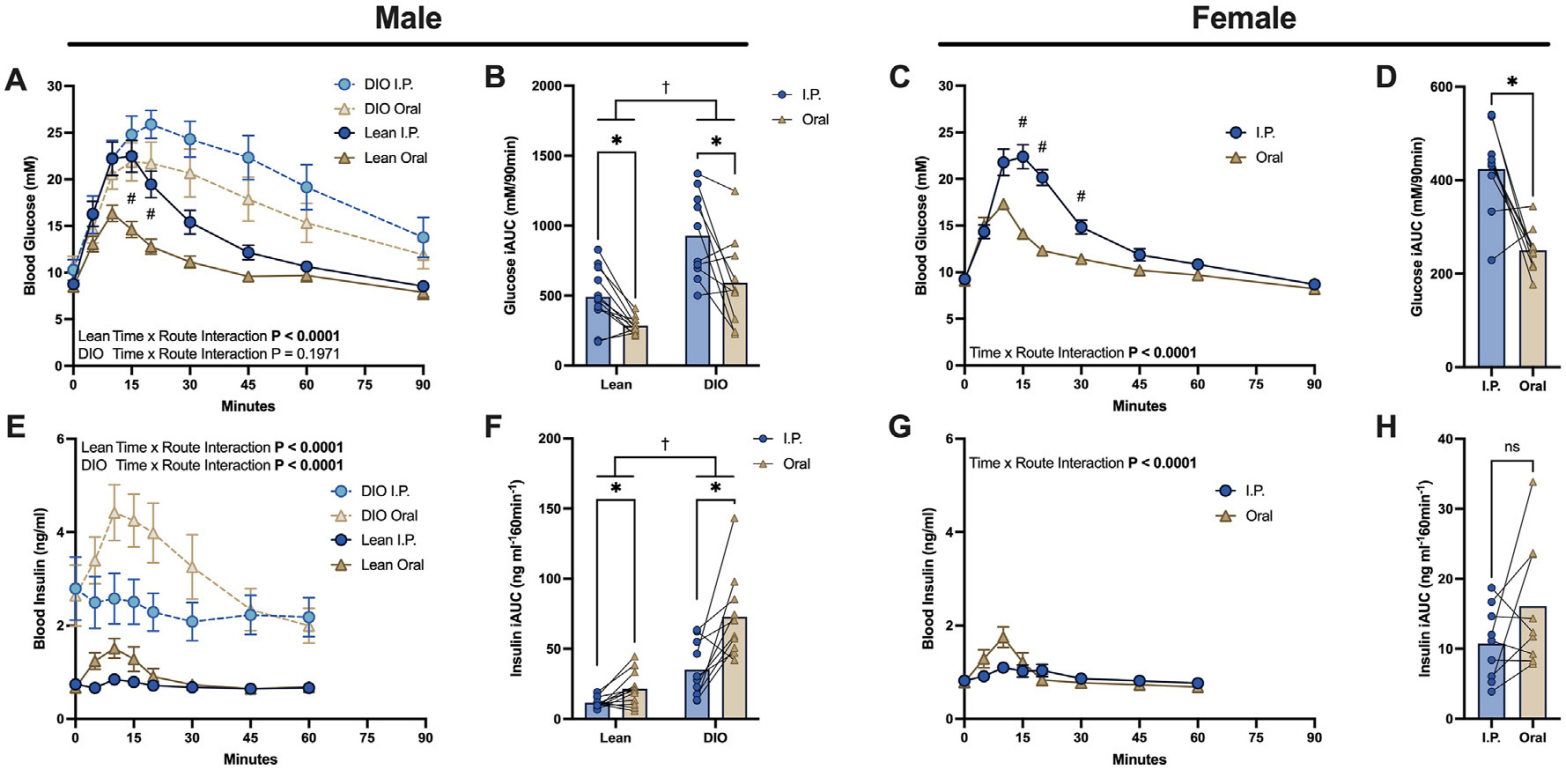
**Comparison of glucose and insulin curves during a GTT after I.P. or oral glucose administration.** In lean (n = 12) and DIO (n = 10) male mice (A) blood glucose and (E) insulin curves during a GTT and the incremental area under the curve of (B) blood glucose and (F) blood insulin analysed by individual Wilcoxon matched-pairs tests adjusted for multiple comparisons and 2-way ANOVA to test for a main effect of diet. In lean, female mice (C) blood glucose and (G) insulin curves during a GTT and the incremental area under the curve of (D) blood glucose and (H) blood insulin analysed by Wilcoxon matched-pairs tests, n = 9. ∗P < 0.05. †P < 0.05 for a main effect of diet. #P < 0.05 comparing between oral and I.P. routes at individual timepoints over the GTT. Values are means ± SE or means with all data points.

**Figure 2:**
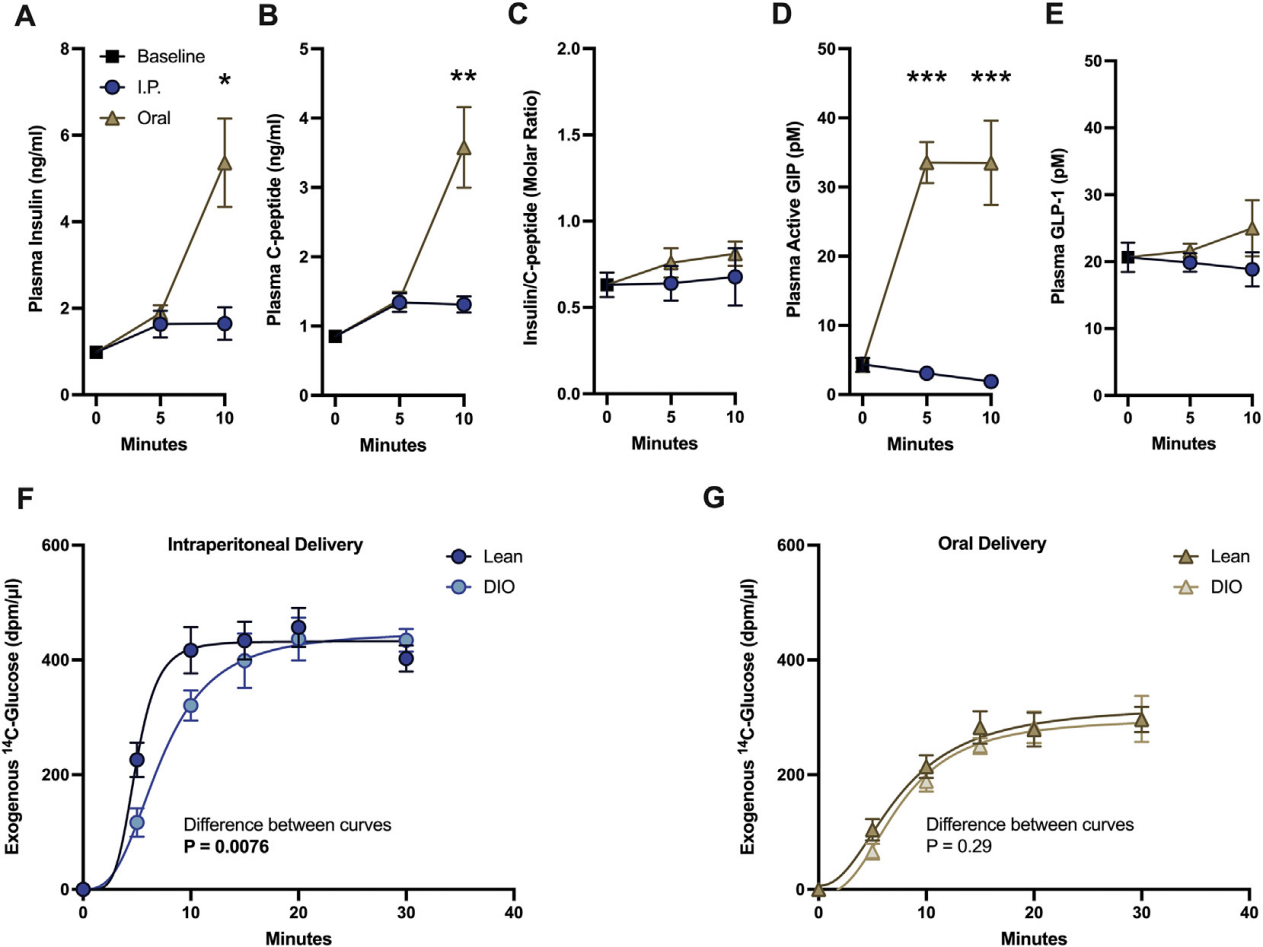
**The early incretin response and rate of appearance of exogenous glucose are affected by the route of glucose administration during a GTT.** In lean male mice, cardiac plasma (A) insulin, (B) c-peptide, (C) the insulin/c-peptide molar ratio and (D) active gastric inhibitory polypeptide, n = 8, and (E) c-terminal glucagon-like peptide 1, n = 6–8, at baseline and 5 and 10 min after glucose administration. Analysed by individual Mann–Whitney U tests at 5 and 10 min, corrected for multiple comparisons. The appearance of exogenous 14C-glucose during a GTT comparing male lean and DIO mice after (F) I.P. and (G) oral delivery as analysed by fitting a sigmoidal curve and comparing absorption half-life, n = 6–8. ∗P < 0.05, ∗∗P < 0.005, ∗∗∗P < 0.0005. Values are means ± SE.

### 2.3 Plasma and blood analysis

Whole-blood insulin was measured using a commercial insulin ELISA kit (Ultra Sensitive Mouse Insulin ELISA Kit, Crystal Chem, #90080) with some modifications to the kit protocol. Prior to the GTT, 95 μl of sample diluent was added to each of the wells in the plate and the plate was kept on ice. During the GTT, for each mouse for each time point, 5 μl of blood was carefully pipetted from the tail-tip into one of the wells of the ELISA plate using a positive displacement pipette (Microman E, 3–25 μl, Gilson) while the plate was kept on ice. Following the GTT, the wide-range standards were added and the plate was incubated at 4 °C overnight (∼18 h). The normal kit protocol was then followed with the addition of 2 washing steps for the initial wash (from 5 to 7). To test the linearity of blood insulin to plasma insulin, prior to euthanasia, blood was taken by cardiac puncture in anesthetized mice into an EDTA tube. A 5 μl aliquot of this blood was added directly to the ELISA plate in duplicate. Plasma was then separated by centrifugation (1,000 g at 4 °C for 10 min) and added in duplicate to the same plate and the protocol above was followed. Plasma c-peptide (#90050), active gastric inhibitory polypeptide (GIP, #81511), and corticosterone (#80556) were measured by ELISAs from Crystal Chem following manufacturer's instructions. Plasma glucagon-like peptide 1 (GLP-1) was measured using a c-terminally directed radioimmunoassay [5] modified for the measurement of mouse GLP-1. Thus, 100 μl aliquots of plasma were mixed with 200 μl PBS containing in addition 6% recombinant human serum albumin and extracted in 70% (final) ethanol.

### 2.4 Stable-isotope GTT and GCMS

A siGTT was performed on a separate cohort of male, 14-16 week-old chow-fed mice following a previously described protocol [6] with some modifications. Briefly, 25 mg of 2-^2^H (M1) and 25 mg of 6,6-^2^H (M2) glucose (Cambridge Isotope Laboratories) dissolved in 200 μl sterile saline were administered to mice by either I.P. injection or oral gavage at 14:00 after a 6-h fast. A 20 μl aliquot of blood was collected into EDTA tubes before and 10, 20, 30, 60, and 90 min following stable glucose administration. The blood was centrifuged at 1,000 *g* at 4 °C for 10 min and plasma was collected. A 5 μl aliquot of plasma was added to 135 μl of methanol, and the sample was left to precipitate on ice for 30 min. Samples were centrifuged at 9,400 g for 3 min at 4 °C and 50 μl of supernatant was taken and transferred into gas chromatography (GC) vials. Extracts were evaporated under a stream of nitrogen until they completely dried. Derivatization was performed using MOX-BSTFA derivatizing agent by a robotic multipurpose sampler using a standard protocol. Briefly, 12.5 μl of MOX (2% solution of methoxyamine–hydrogen chloride in pyridine) was added to the dried extract and mixed for 1 h at 45 °C. Subsequently, 12.5 μl of BSTFA (N, O-bis(trimethylsilyl)trifluoroacetamide + 1% trimethylsilyl chloride) was added to the vial. Before injection, 50 μl of hexane containing 5 mg/l of 4,4′-dibromooctafluorobiphenyl was added as an injection standard. The Pegasus BT GC-TOFMS (LECO, St. Joseph, USA) system was used for automatic derivatization, chromatography separation and mass detection equipped with an MPS autosampler (Gerstel, Mülheim an der Ruhr, Germany). Chromatographic separation was performed on a 30 m MS5 chromatographic column. The temperature gradient ranged from 40 °C to 340 °C over 19 min. Detection was made in the full scan mode from 50 to 750 Da. The mass spectrometer was equipped with an electron ionization source with standard 70eV fragmentation. The isotopic pattern for each glucose isotope was validated by injecting serial dilutions of each glucose isotope to determine isotopic patterns over different concentration ranges. The characteristic isotopic difference for 2-^2^H glucose was between 161 and 160 ions and for 6,6-^2^H glucose was between 321 and 319 ions and therefore 4 ions (160, 161, 319, and 321) were isolated from the spectra and integrated using an SMC in-house script. The fractional abundance of each glucose isotope was calculated in Microsoft Excel. Exogenous and endogenous glucose was calculated by multiplying blood glucose concentration determined by a handheld glucometer (Contour XT, Bayer) by the fractional abundance of glucose isotopes as described [6]. Hepatic glucose cycling was calculated by comparing plasma fractional enrichment of 2-^2^H and 6,6-^2^H isotopes as described [7].

### 2.5 Statistical analysis

Glucose and insulin time-courses were analysed by repeated-measures two-way ANOVA for a time, route of administration interaction. If a significant interaction was found Sidak *post hoc* test was performed on all timepoints comparing between routes of glucose administration and adjusted for multiple comparisons. AUC was calculated using the trapezoidal method, incremental AUC was calculated by determining trapezoidal area subtracted from the lowest value for each mouse. To determine statistical significance, data were tested for normality using the Anderson-Darling test and equal variance by Spearman's rank correlation test, and a suitable parametric or non-parametric test was performed as described in the figure legends. To compare glucose appearance, absorption half-life was compared by fitting data to a sigmoidal curve and comparing IC50 (Figure 2F and G). Data were analysed using GraphPad Prism (GraphPad, version 9).

## 3 Results and discussion

### 3.1 I.P. glucose administration leads to an increased glucose excursion and a largely absent insulin excursion compared to oral administration

The glucose tolerance test is a commonly used tool in metabolic research to investigate glucose metabolism in mice. In this study, we demonstrated several important differences in glucose handling during GTTs performed either by the oral or I.P. route, which may have an impact on the interpretation of GTT results. There was no difference in body composition or fasting glycemia between mice receiving oral or I.P. injection (Table 1), although, as expected, DIO mice had substantially more fat mass, higher body weight, and higher fasting blood glucose levels than lean mice. I.P. glucose administration led to a far higher glucose excursion than oral-dosed mice in both lean and DIO male mice (Figure 1A and B) and in lean female mice (Figure 1C and D). The lower glucose excursion in orally-dosed mice was accompanied by a ∼2-fold increase in blood insulin concentration, peaking 10 min after glucose administration and returning to basal levels 30–45 min after (Figure 1E-H). In comparison, a glucose-induced rise in blood insulin concentrations after I.P. glucose administration was largely absent in male mice (Figure 1E and F), while the peak was reduced in female mice (Figure 1G), although the incremental area under the curve (iAUC) for insulin was not significantly different between I.P. and orally dosed female mice (Figure 1H).

**Table 1:** Average weight, body composition, and fasting glycaemia of mice prior to GTT.

	Before I.P. injection	Before oral injection	Effect of route (P-value)
**Male lean mice (n = 12)**			
Body weight (g)	32.4 ± 0.7	32.5 ± 0.6	0.55
Lean mass (g)	22.5 ± 0.7	22.0 ± 0.5	0.27
Fat mass (g)	5.0 ± 0.6	5.4 ± 0.6	0.14
Fasting blood glucose (mM)	8.8 ± 0.3	8.5 ± 0.4	0.49
**Male DIO mice (n = 10)**			
Body weight^†^	44.2 ± 1.4	44.9 ± 1.2	0.54
Lean mass	22.5 ± 0.7	22.3 ± 0.5	0.46
Fat mass^†^	13.6 ± 1.2	14.2 ± 1.0	0.44
Fasting blood glucose (mM)^†^	10.3 ± 1.1	10.8 ± 0.9	0.31
**Female lean mice (n = 9)**			
Body weight	28.2 ± 0.9	28.2 ± 1.0	1.00
Lean mass	16.7 ± 0.2	16.7 ± 0.3	0.63
Fat mass	6.0 ± 0.6	5.9 ± 0.6	0.90
Fasting blood glucose (mM)	9.3 ± 0.3	9.1 ± 0.4	0.36
**Male lean mice siGTT (n = 9)**			
Body weight	29.4 ± 0.5	28.9 ± 0.8	0.58
Fasting blood glucose (mM)	8.6 ± 0.4	8.4 ± 0.3	0.75

Bodyweight and body composition measured by MRI 24 h prior to the GTT and fasting blood glucose in lean and DIO male mice and lean female mice, randomized to I.P. or oral glucose administration. Analysed by paired t-tests comparing between routes and a 2-way ANOVA for the main effect of diet comparing male lean and DIO mice. Body weights for lean male mice 24 h prior to the siGTT and fasting blood glucose as analysed by Student's t-test. Values are means ± SE. †P < 0.005 main effects of diet comparing lean and obese male mice.

Although some studies report increased plasma insulin concentrations following I.P. glucose injection in mice [1,[8], [9], [10]], the largest changes often occur very rapidly, around 2 min after injection [9,10] and then quickly disappear (similar to the insulin response after an intravenous GTT in mice [11]) or ∼60 min after the start of the GTT [1], by which time much of the glucose bolus has already been cleared. There are also many examples in which I.P. injection of glucose leads to a negligible or very little rise in insulin concentrations in mice [[12], [13], [14], [15], [16]]. This discrepancy may be due to sampling times, as an early rise in insulin or first-phase insulin secretion may be missed. Anaesthesia may also be a factor; this study was performed in conscious mice as, depending on the anaesthetic used, anaesthesia can both potentiate or negate glucose-induced rises in insulin during an oGTT [17]. Alternatively, fasting time may play a role whereby a rise in insulin level may more easily be detected in overnight fasted animals due to lower fasting insulin concentrations. However, due to the impact of extended fasting on body weight and lean mass in mice [18,19], we would not recommend overnight fasting before a GTT. It is important to consider that even shorter fasts, such as the 6-h fast used in this study, can lead to a significant body weight loss compared to non-fasted animals [19]. However, a 6-h fast was shown to be the ideal duration for discriminating differences in glucose tolerance between chow- and HFD-fed mice [1]. Another consideration is the choice of mouse strain used. In the present study, the C57BL/6NTac mouse strain was chosen as it has a more robust insulin secretion response during a GTT compared to the C57BL/6J [20] or C57BL/6NJ [21] strains. These latter strains harbour a deletion in the nicotinamide nucleotide transhydrogenase gene which has been shown to affect insulin secretion during a GTT [22].

### 3.2 Higher insulin response in orally-dosed mice is associated with a robust GIP response which is absent after I.P. glucose injection

To further investigate the insulin response acutely after glucose administration, we examined circulating concentrations of insulin, c-peptide, and incretin hormones acutely following glucose administration in a separate cohort of mice. Similar to the previous cohort, plasma insulin was substantially elevated only in orally dosed mice 10 min after glucose delivery (Figure 2A). C-peptide showed a very similar pattern (Figure 2B), and the insulin/c-peptide molar ratio was similar between groups suggesting similar insulin clearance between oral and I.P. dosed mice (Figure 2C). Insulin concentrations from this cohort were substantially higher than those in the previous cohort (Figure 1E) which may be due to sampling directly from the heart. Plasma levels of active GIP were substantially elevated after oral but not I.P. glucose administration and preceded rises in insulin and c-peptide (Figure 2D). Measured levels of plasma GLP-1 were not significantly elevated from baseline after either oral or I.P. glucose administration (Figure 2E) which may be due to its rapid *in vivo* degradation which prevents accurate measurements, even with c-terminal-based assays [23]. Inhibition of DPP-4 and neutral endopeptidase *in vivo* may therefore be required for accurate measurement of GLP-1 secretion in the mouse [23]. Overall, our results suggest that most of the insulin response after glucose administration in mice is driven by the incretin response rather than classical glucose-stimulated insulin secretion. A similar result was found when c-peptide secretion was compared between the intravenous GTT and oGTT in mice [24] and was comparable to what occurs in humans in which the incretin response has been calculated to be responsible for up to 80% of post-glucose insulin secretion [25]. However, classical glucose-stimulated insulin secretion may partly be responsible for substantial fasting hyperinsulinemia measured in the DIO mice (Figure 1E), to normalise glycaemia in the presence of systemic insulin resistance.

### 3.3 Exogenous glucose appearance into circulation is reduced in obese mice compared to lean mice after I.P. but not oral glucose injection

The substantially lowered blood glucose excursion after oral glucose administration may be the result of a combination of factors. These factors include an increase in insulin-mediated glucose disposal, a decrease in endogenous glucose production, primarily by the liver and, a potentially lower dose of glucose reaching circulation due to gradual gastric emptying and delayed glucose absorption in the GI tract. It has been calculated that in mice ∼40% of an exogenous glucose bolus remains in the stomach 60 min after intragastric administration; however, the majority (90%) of glucose that reaches the duodenum is absorbed [26]. One assumption about the ipGTT is that glucose injected into the peritoneal cavity drains into portal circulation [27] at a similar rate regardless of visceral adiposity. However, we report that in obese mice, I.P. administration of glucose leads to a reduced rate of exogenous glucose appearance into circulation as compared to lean mice (Figure 2F), which is not present after oral gavage (Figure 2G). Differences in the rate of exogenous glucose appearance between groups complicate the interpretation of ipGTTs and these differences are likely due to direct injection into visceral fat, reduced diffusion, or reduced local absorption by capillaries in the peritoneal cavity.

### 3.4 Hepatic glucose production is suppressed only after oral glucose administration

**Figure 3:**
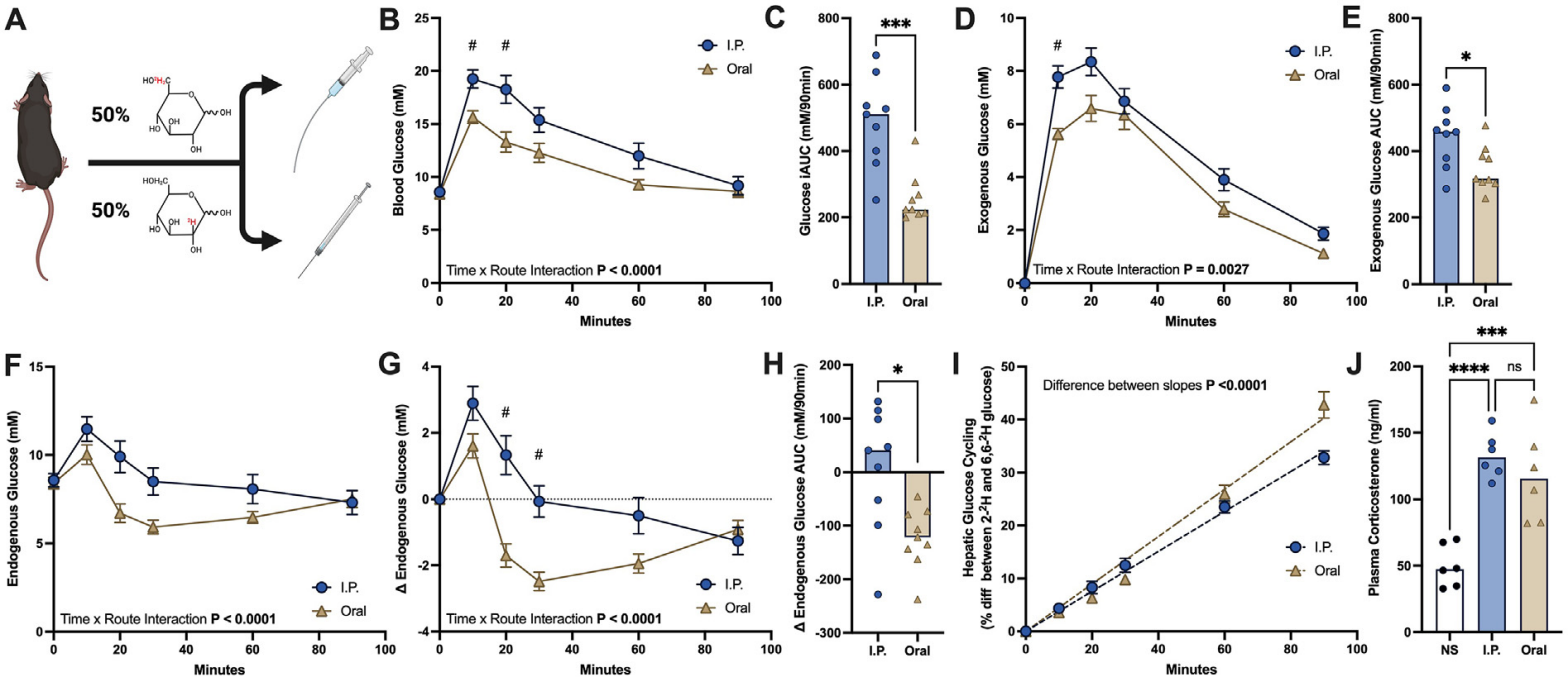
**The route of glucose administration during a GTT affects endogenous glucose production and hepatic glucose cycling.** (A) Schematic of stable-isotope labelled GTT performed in lean, male mice. During the GTT (B) blood glucose and (C) the blood glucose iAUC, (D) exogenous glucose and (E) the exogenous glucose AUC, (F) endogenous glucose, (G) change in endogenous glucose from baseline, and (H) the AUC for the change in endogenous glucose from baseline. AUCs analysed by the Mann–Whitney U test. (H) Rate of hepatic glucose cycling during the stable-isotope labelled GTT, analysed by comparing slopes after linear regression, n = 9. (J) Plasma corticosterone following I.P. or oral GTT (120 min after glucose administration) and in non-stressed mice (NS) taken at the same time of day, n = 6, analysed by one-way ANOVA with Tukey post-hoc test. ∗P < 0.05, ∗∗∗P < 0.0005, ∗∗∗∗P < 0.0001. #P < 0.05 comparing between oral and I.P. routes at individual timepoints over the GTT. Values are means ± SE or means with all data points.

We next performed a siGTT in a separate cohort of lean, male mice to examine differences in exogenous and endogenous glucose during a GTT between I.P. and oral routes of glucose administration (Figure 3A). Body weight was similar between mice randomized to receive I.P. or oral glucose administration (Table 1). During the siGTT, blood glucose showed a similar pattern to the previous cohort of mice with oral gavage, leading to a significantly reduced glucose excursion compared to I.P. administration (Figure 3B and C). Exogenous glucose peaked at a lower level after oral glucose administration compared to the I.P. route, suggesting a reduced dose of glucose entering circulation (Figure 3D and E), comparable to what was reported in the first cohort of mice utilising ^14^C labelled glucose (Figure 2F and G). Endogenous glucose was increased in both oral and I.P. dosed mice 10 min after glucose administration (Figure 3F), presumably due to stress-induced liberation of hepatic glycogen [28]. Endogenous glucose was then suppressed in mice that received oral glucose, throughout the GTT, with a nadir of a ∼2.5 mM decrease in endogenous blood glucose from baseline observed 30 min after glucose administration (Figure 3G). In comparison, endogenous glucose production was not suppressed in I.P. dosed mice (Figure 3F–H). Hepatic glucose cycling, a measure of the combined, opposing activities of glucokinase and glucose-6-phosphatase [6,7], was higher in oral versus I.P. dosed mice (Figure 3I), suggesting an increased rate of hepatic glucose uptake in orally-dosed mice as endogenous glucose production was lower (Figure 3H). The failure to suppress endogenous glucose production and the reduced hepatic glucose uptake after I.P. glucose administration is likely the result of a lack of insulin response (Figure 1, Figure 2A), as both are hallmarks of insulin action in the liver [29].

### 3.5 Changes in whole-body insulin sensitivity may not be detected by the ipGTT

The translation of GTTs performed in mice to those performed in humans has recently been discussed [3,28], with several important species differences impacting glucose and insulin kinetics. One potentially important difference between mice and humans is the contribution of insulin-mediated and non-insulin-mediated pathways to clear a glucose load, with a recent study arguing that the majority of glucose disposal after an oral glucose load in mice is cleared by non-insulin-dependent pathways [28]. Our data largely agree with this study as glucose was cleared in I.P-dosed mice (Figure 1A-D) without an elevation in circulating insulin (Figure 1E and F), although less rapidly than after oral administration. The ability of glucose to stimulate its own disposal is termed glucose effectiveness and may play a larger role in mice than in humans [28]. Nevertheless, in humans, glucose effectiveness still plays a major role and is thought to be responsible for up to half of glucose disposal during a GTT [30]. Although relative insulin sensitivity cannot be directly inferred from a GTT, changes in glucose clearance and circulating insulin levels can be determined. Therefore, taking DIO mice as an example, when mice display an increased glucose excursion during a GTT (Figure 1A), indicating relative glucose intolerance, as well as a similar or higher circulating insulin excursion than control mice (Figure 1E and F), it can be inferred that the DIO mice have reduced whole-body insulin sensitivity or reduced glucose effectiveness, or a combination of the two. However, when peripheral insulin levels are not increased, as we report after I.P. glucose injection (Figure 1E), no assumptions can be made about insulin sensitivity as the test is only measuring glucose effectiveness. Therefore, the oGTT, and not necessarily the ipGTT, provides relevant information regarding whole-body glucose handling in a more physiological setting than the hyperinsulinemic-euglycemic clamp. Nevertheless, the ipGTT can still be a useful tool when the oGTT is also performed on the same mice as a means of determining the contribution of the GI tract, as has been conducted to assess the effect of incretin hormones on glucose metabolism [[31], [32], [33], [34]]. Similarly, if a difference in gastric emptying or glucose absorption is suspected, then an ipGTT may be performed in addition to an oGTT to standardise exogenous glucose appearance into circulation (although care must be taken when comparing between mice with large differences in body composition as previously mentioned).

### 3.6 Measuring insulin in the blood is a good alternative to plasma to reduce the volume of blood samples taken during a GTT

**Figure 4:**
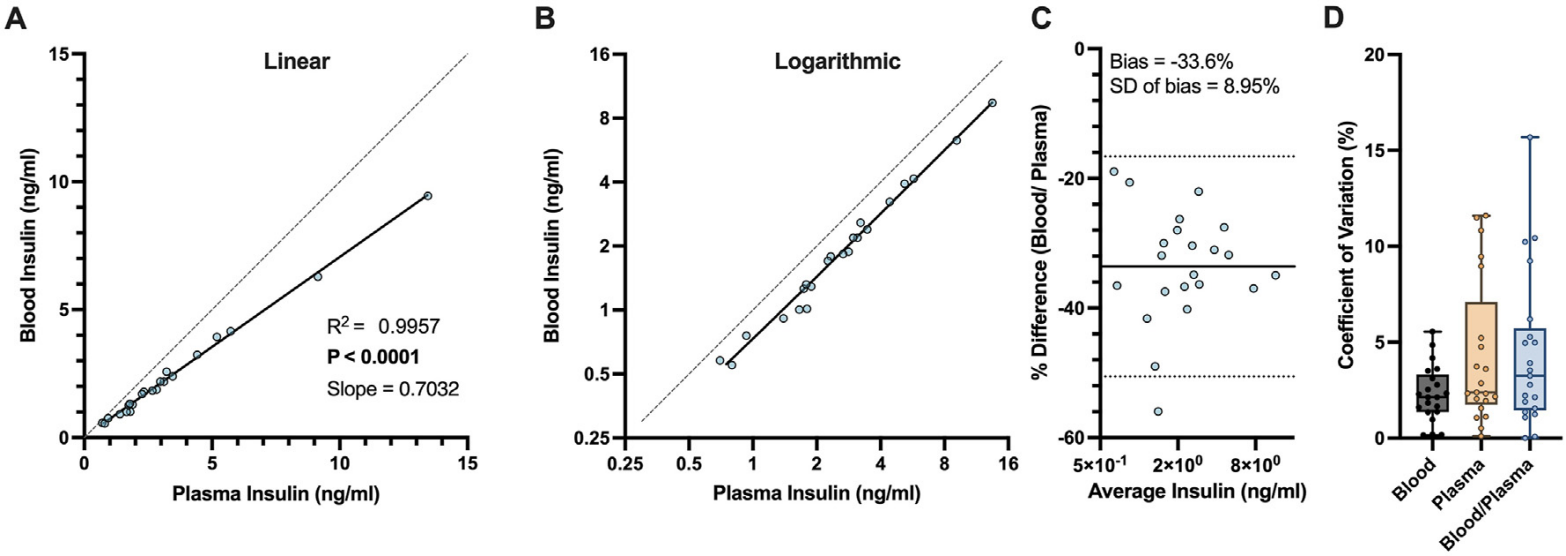
**Measuring insulin from whole blood gives comparable results to plasma.** Correlation between plasma and blood insulin measurements in the (A) linear and (B) logarithmic scale, analysed by simple linear regression, n = 22. (C) Bland–Altman plot comparing the blood assay to the plasma assay, solid line is the mean of bias and dashed lines are the 95% limits of agreements. (D) Intra-assay variation between technical duplicates when measuring insulin from whole-blood and plasma and inter-assay variation when comparing between normalised whole blood (values divided by the slope in Figure 3A) and plasma, analysed by the Kruskal–Wallis test, n = 21.

Another element in assessing glucose tolerance is the effect of stress, as GTTs performed in conscious mice will be associated with a considerable stress response, unlike in humans [28]. Levels of the stress hormone, corticosterone did not differ between routes of administration following the GTT (Figure 3J). However, plasma corticosterone was increased compared to mice that did not undergo a GTT. As recently discussed [28], a large part of the stress response likely comes from manipulating the tail to collect blood, and therefore GTT procedures in which smaller blood samples are taken may reduce stress-induced rises in blood glucose. We report that insulin can be reliably measured from small (as low as 5 μl) samples of blood by ELISA (as measured in previous publications [35,36]). Whole blood insulin shows a strong linear correlation with plasma insulin (Figure 4A) at both low and high concentrations (Figure 4B) with whole-blood insulin values recorded as ∼70% of plasma values (Figure 4C) likely due to dilution of insulin in whole-blood by the non-plasma fraction. Intra-assay variation between technical duplicates was not significantly different between assays performed on whole-blood or plasma (median CV was 2.1% for whole-blood, 2.4% for plasma, Figure 4D). Similarly, inter-assay variation was similar when comparing normalised whole-blood (divided by the slope in Figure 4A: 0.7032) to plasma (median CV was 3.3%, Figure 4D). These data suggest that measuring circulating insulin levels from whole blood by ELISA provides comparative results to plasma which substantially reduces the amount of blood needed to measure insulin during a GTT.

### 3.7 Conclusion

The route of glucose administration has a large impact on glucose handling during a GTT due to multiple factors including the rate of glucose absorption, the amount of glucose reaching circulation, rise in insulin levels, suppression of endogenous glucose production, and differences in insulin-mediated glucose disposal. In considering these differences, we recommend that unless investigating the effect of the GI tract (comparing oGTT with ipGTT), when performing a GTT in mice, glucose should be administered orally to reduce the chance of artifacts originating from a non-physiological route of administration and to better model postprandial glucose metabolism.

## Author contributions

Lewin Small: conceptualization, formal analysis, investigation, methodology, visualization, writing – original draft and writing review & editing. Amy Ehrlich: investigation. Jo Iversen: investigation. Stephen P Ashcroft: investigation. Kajetan Trošt: investigation and methodology. Thomas Moritz: methodology and supervision. Bolette Hartmann: investigation and methodology. Jens J. Holst: methodology. Jonas T Treebak: writing – review & editing. Juleen R Zierath: supervision and writing – review & editing. Romain Barrès: funding acquisition, supervision, and writing, review and editing of manuscript.
